# Engineering a self-eliminating transgene in the yellow fever mosquito, *Aedes aegypti*

**DOI:** 10.1093/pnasnexus/pgac037

**Published:** 2022-03-30

**Authors:** Keun Chae, Chanell Dawson, Collin Valentin, Bryan Contreras, Josef Zapletal, Kevin M Myles, Zach N Adelman

**Affiliations:** Department of Entomology, Texas A&M University, College Station, TX 77843, USA; Department of Entomology, Texas A&M University, College Station, TX 77843, USA; Department of Entomology, Texas A&M University, College Station, TX 77843, USA; Department of Entomology, Texas A&M University, College Station, TX 77843, USA; Department of Industrial and Systems Engineering, Texas A&M University, College Station, TX 77843, USA; Department of Entomology, Texas A&M University, College Station, TX 77843, USA; Department of Entomology, Texas A&M University, College Station, TX 77843, USA

**Keywords:** *Aedes aegypti*, genetically modified organisms, single-strand annealing, genome editing, DNA repair

## Abstract

Promising genetics-based approaches are being developed to reduce or prevent the transmission of mosquito-vectored diseases. Less clear is how such transgenes can be removed from the environment, a concern that is particularly relevant for highly invasive gene drive transgenes. Here, we lay the groundwork for a transgene removal system based on single-strand annealing (SSA), a eukaryotic DNA repair mechanism. An SSA-based rescuer strain (*kmo^RG^*) was engineered to have direct repeat sequences (DRs) in the *Aedes aegypti kynurenine 3-monooxygenase* (*kmo*) gene flanking the intervening transgenic cargo genes, *DsRED* and *EGFP*. Targeted induction of DNA double-strand breaks (DSBs) in the *DsRED* transgene successfully triggered complete elimination of the entire cargo from the *kmo^RG^* strain, restoring the wild-type *kmo* gene, and thereby, normal eye pigmentation. Our work establishes the framework for strategies to remove transgene sequences during the evaluation and testing of modified strains for genetics-based mosquito control.

Significance Statement In order to prevent mosquito-transmitted diseases, approaches based on genetic control of vector populations are being developed. However, many such strategies are based on highly invasive, self-propagating transgenes that can rapidly spread the trait into native populations with ecological consequences difficult to predict and potentially impossible to reverse. Although various confinable, self-exhausting approaches are being developed to mitigate unintended issues in genetics-based population control, many of those focus on the process of drive itself, rather than the transgene components, which may remain in the population for extended periods of time. Here, we show that an SSA-based transgene removal system decreases the stability of transgenes from the *Ae. aegypti* genome, suggesting a novel pathway for engineering safety features into approaches for genetic control of vector mosquito populations.

## Introduction

To control vector mosquito populations, genetics-based control methods have been proposed based on Sterile Insect Technique (SIT), Release of Insects carrying a Dominant Lethal (RIDL) and/or gene drive (1–4). In gene drive approaches, the modified organism carries 1 or more genetic elements that permit the rapid introgression of the genetic trait into the target species population via super-Mendelian inheritance (2). The development of the CRISPR/CRISPR-associated protein 9 (Cas9) system dramatically accelerated homing gene drive strategies in malaria and dengue transmitting mosquitoes (5–10). CRISPR-based homing gene drive approaches have been proposed that could permanently alter the genomes of disease vectors for the purposes of either population suppression or population replacement (rendering vectors unable to transmit pathogens) (1, 11). With this come concerns related to releasing genetically modified organisms (GMOs), in terms of both health and ecological safety (12, 13). For example, a gene drive transgene could potentially invade related nontarget populations, and given their invasive, self-propagating nature, it may be impossible to remove such transgenic material once out in the field. As potential hazards to ecosystems are still uncertain (14–18), the ability to limit and/or remove a gene drive transgene is perceived as a major factor in the potential acceptability of these technologies (19–21). Confinable gene drive strategies in a split or daisy-chain system have been proposed to eliminate unwanted invasion to nontarget populations (10, 22), while genetic technologies such as CATCHA, e-CHACRs, ERACRs, and the anti-CRISPR AcrIIA4 protein have shown promise to limit the activity of gene drive transgenes (23–25). While these approaches could limit the process of gene drive, removing the transgenes themselves is not simple and in many cases would require remediation in the form of mass release of wild-type insects. We recently proposed several technological designs that take advantage of naturally occurring DNA repair mechanisms potentially capable of deleting transgenes scarlessly from the genome of a gene drive mosquito and predicted to reverse the invasion of a gene drive transgene back to the wild-type (26).

**Fig. 1.**
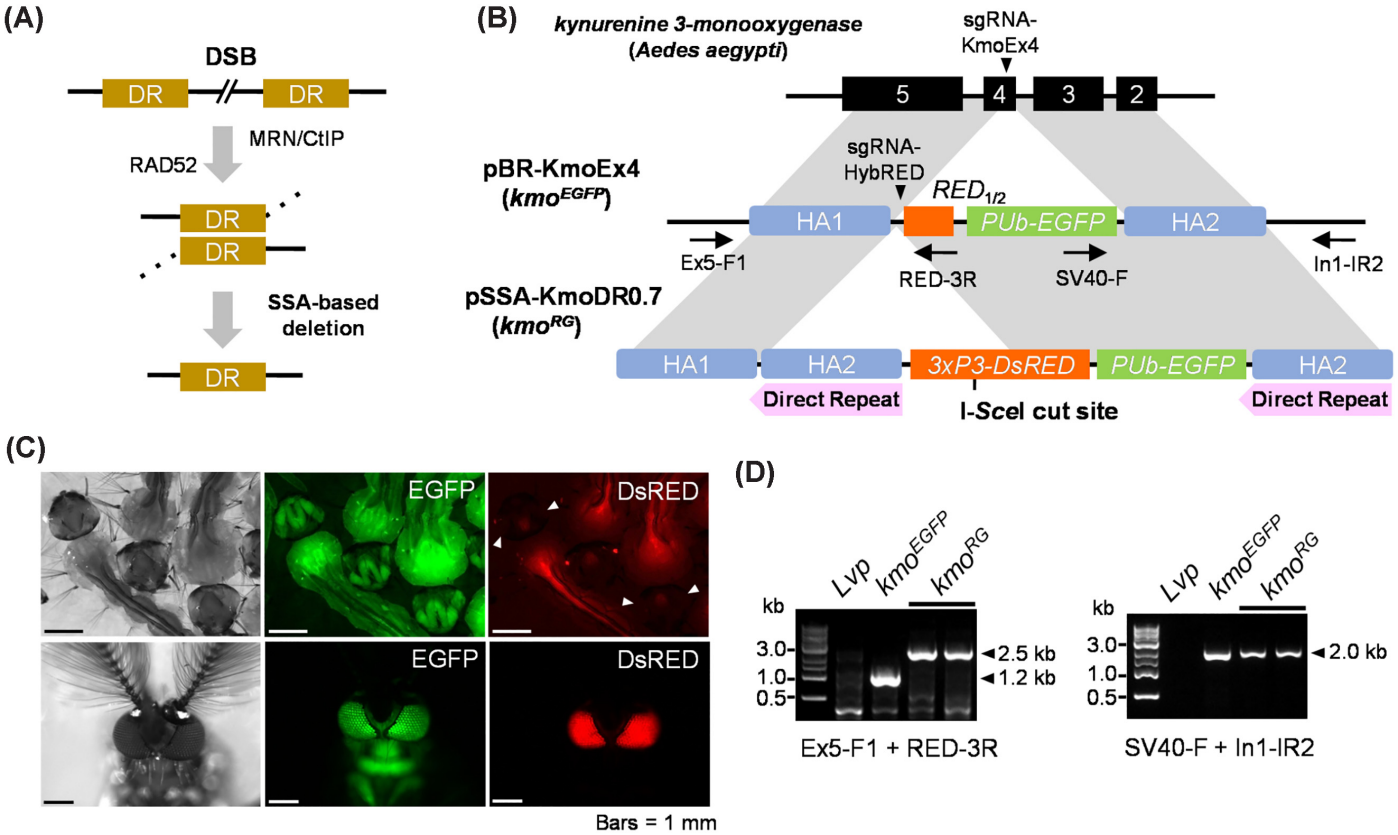
*Aedes aegypti* transgenic strains for SSA-based transgene elimination. (A) Schematic representation of the eukaryotic SSA mechanism. The DNA DSBs can be repaired by the SSA pathway in the presence of flanking DR motifs. Following DNA end resection from the DSB site by the MRN (MRE11-RAD50-NBS1)/CtIP complex, 2 DRs are aligned parallelly by RAD52 based upon sequence homology, and then the intervening sequence is degraded. (B) Schematic representation of plasmid constructs pBR-KmoEx4 and pSSA-KmoDR0.7 for the development of stage 1 *kmo^EGFP^* and stage 2 *kmo^RG^* strains, respectively. For pBR-KmoEx4, sgRNA-KmoEx4 was designed to target exon4 of the *Ae. aegypti kmo* gene (Figure S1A, Supplementary Material) and flanking *kmo* sequences (∼0.7 kb) were included as HAs, HA1 (exon4/5) and HA2 (exon2/3). *PUb-EGFP* and *RED*_1/2_ (3'-half of *DsRED*) were interposed between the 2 HAs as transgene cargos. For pSSA-KmoDR0.7, sgRNA-HybRED was created to target to *RED*_1/2_ in the *kmo^EGFP^* strain (Figure S1B, Supplementary Material). The stage 2 *kmo^RG^* strain carries the additional *kmo* exon2/3 (HA2) as the DRs (pink bars) and *3xP3*-driven full-sized *DsRED*, which was modified to contain the I-*Sce*I recognition sequence next to ATG translation start codon. (C) Transgenic *kmo^EGFP^*(top)*and kmo^RG^* (bottom) strain mosquito larvae and adults as viewed under white light, EGFP, and DsRed filters. The *kmo^EGFP^* strain did not show DsRED fluorescent eyes (arrow heads), because it has *RED*_1/2_, a truncated *DsRED* gene. (D) PCR analysis for chromosomal integration of donor plasmid constructs at the *kmo* locus in the transgenic mosquitoes. A total of 2 pairs of PCR primers (horizontal arrows in Fig. 1B; Table S1, Supplementary Material) were utilized to recognize the junction areas between cargo genes and *kmo* genomic sequences outside of HAs.

Mosquitoes, like all eukaryotes, rely on DNA repair systems to process DNA double-strand breaks (DSBs) by mainly 2 pathways; nonhomologous end joining (NHEJ) or homology-directed repair (HDR) (27, 28). In NHEJ, the Ku complex initially binds the DSB site and subsequently recruits the DNA–PKcs/Artemis complex and the XRCC4–DNA Ligase IV complex to repair the broken DNA ends, potentially generating insertions or deletions in the process. In contrast, the HDR pathway can repair DSBs by using a homologous template sequence from a sister chromosome (29, 30). In the latter case, DNA end-resection at the DSB site results in a 3'-single-stranded DNA (ssDNA) tail that allows other necessary factors including the MRN/X complex, RAD51, and BRCAs to be recruited for strand invasion during the repair process (31, 32). Interestingly, when DSB-induced ssDNA resection occurs between 2 identical sequences, known as direct repeat sequences (DRs), the single-strand annealing (SSA) pathway allows the DRs to be annealed and triggers the intervening sequences to be deleted (33, 34) (Fig. 1A). Our previous work demonstrated that this highly deleterious form of repair occurs readily in *Aedes aegypti* mosquitoes (35). Thus, we hypothesized that if a series of transgenes is engineered with flanking DR sequences, these could be subsequently deleted via the SSA pathway when required, while simultaneously resulting in the restoration of the wild-type genotype.

Here, we present a proof-of-concept genetic system as a prelude to self-eliminating transgene technologies (26) to preprogram the elimination of transgene cargos in the mosquito *Ae. aegypti* by taking advantage of the SSA pathway. We used site-specific recombination to insert 2 transgenes within the *Ae. aegypti kmo* locus. Endonuclease-driven DSBs at 1 of the reporter genes triggered both NHEJ and SSA-based repair. Most importantly, the SSA pathway removed all exogenous cargo and flawlessly restored the wild-type gene and the normal eye pigmentation phenotype from the transgenic, white-eyed mosquitoes. Multigenerational tests indicated that the rate of SSA-based transgene elimination assisted by natural selection substantially increased the number of wild-type individuals in the test populations. The SSA-based self-eliminating transgene system developed in this study provides the basis for potential rescue strategies for transgenesis-based mosquito population control.

## Results

To establish an SSA-based transgene removal system in *Ae. aegypti*, we performed site-specific insertion of transgene sequences targeting the *kynurenine 3-monooxygenase* (*kmo*) gene as the recipient locus in a 2-stage process (Fig. 1B; Table S1, Supplementary Material). For the 1st stage, a *polyubiquitin-EGFP* (*PUb-EGFP*) reporter cassette and the 3'-portion of the *DsRED* (*RED*_1/2_) gene were flanked by homology arm (HA) sequences (771 bp from exon4/5 for HA1 and 684 bp from exon2/3 for HA2) with DSB induction triggered by Cas9 complexed with a single synthetic guide RNA (sgRNA-KmoEx4; Figure S1A and Table S2, Supplementary Material). EGFP^+^ individuals were used to establish a strain we refer to as *kmo^EGFP^*. In the second stage, a new sgRNA (sgRNA-HybRED) was designed to recognize the boundary sequence of the *RED*_1/2_ in the *kmo^EGFP^* strain (Figure S1B, Supplementary Material), with the new transgene sequences flanked by corresponding HAs (Fig. 1B). The result of this integration was that the HA2 region was duplicated next to HA1, creating DRs of approximately 700 bp that could be utilized by the SSA pathway. This 2-stage process was necessary to prevent competition in repair between the 2 HA2 motifs, as use of the HA2 in proximity to HA1 could result in repair of the *kmo* gene with no integration of the transgenes. As expected, the stage 2 *kmo^RG^* mosquitoes displayed DsRED fluorescence in the eyes (36), EGFP fluorescence in the body (37), and white-colored eyes due to loss of *kmo* (38, 39) (Fig. 1C). The site-specific insertion of each cassette was verified by PCR analysis for both *kmo^EGFP^* and *kmo^RG^* strains (Fig. 1D). In order to trigger a DSB in the transgene sequence and initiate SSA, an I-*Sce*I recognition site was included in-frame following the ATG translational start codon of the *DsRED* gene. This position was advantageous in that it could potentially allow the identification of NHEJ-based repair events (DsRED^–^/EGFP^+^/Kmo^–^; referred to as *kmo*^G/Δ4^) in addition to SSA-based events (DsRED^–^/EGFP^–^/Kmo^+^; referred to as *kmo*^+/Δ4^).

**Fig. 2.**
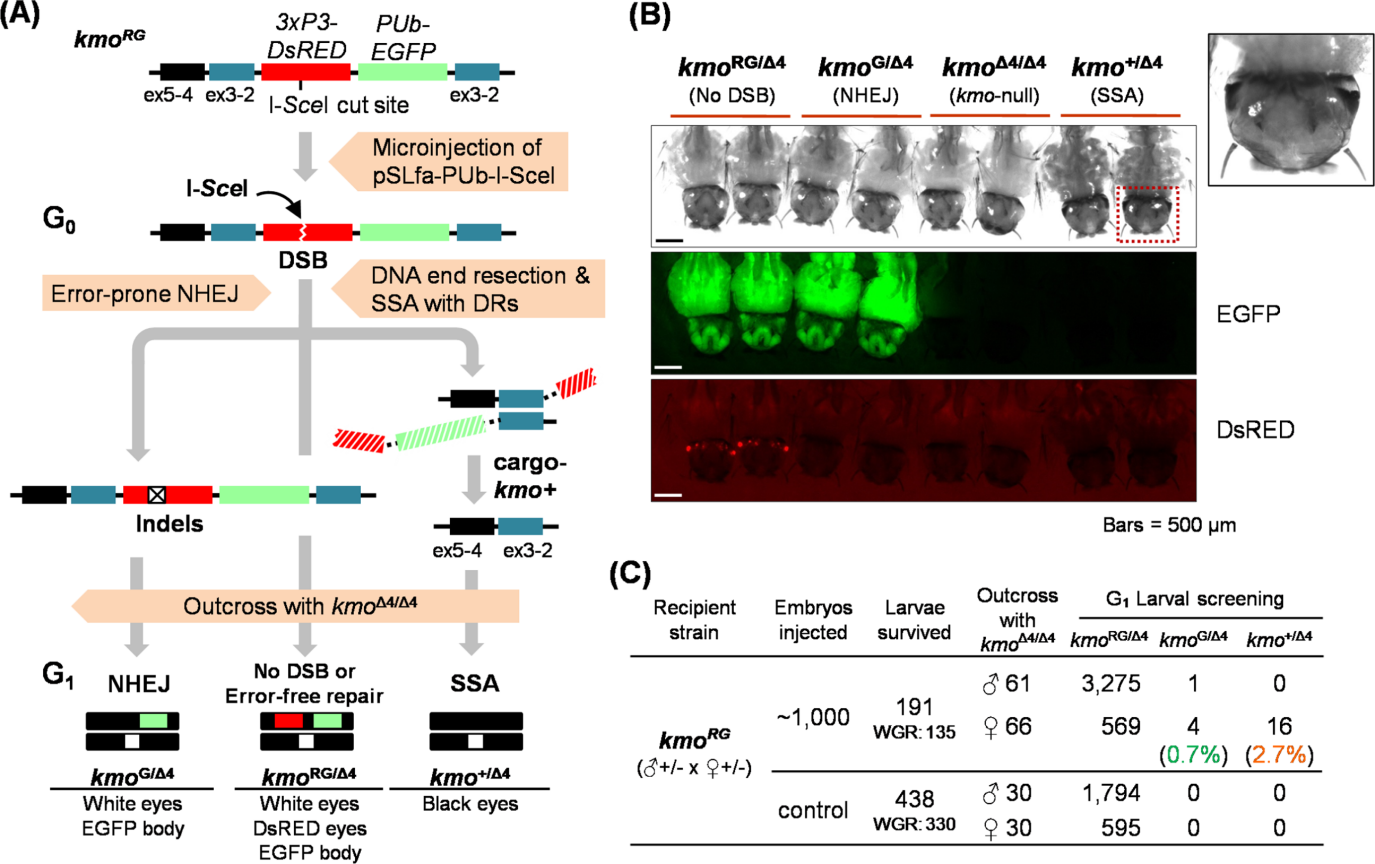
SSA-based transgene elimination triggered by plasmid DNA expressing a HE, I-*Sce*I. (A) Schematic workflow representation of evaluating the SSA-based transgene removal system engineered in the *kmo^RG^* strain. (B) Distinct DNA repair-associated phenotypes in eye pigmentation and marker fluorescence of G_1_ larvae in the SSA test. The insert is a magnified image of black-colored eyes restored by SSA-driven transgene elimination from the targeted *kmo* gene. (C) Summary of the SSA test using a plasmid-based SSA trigger. G_0_ embryos that were not microinjected served as negative controls.

As an initial test of SSA-driven elimination of the transgene in the *kmo^RG^* strain, we microinjected preblastoderm embryos, obtained from a cross between heterozygous *kmo*^RG/Δ4^ parents, with a donor plasmid expressing the homing endonuclease (HE) I-*Sce*I to induce DSB formation in the transgene (Fig. 2A). Only *kmo^RG^* G_0_ survivors, consisting of both homozygous *kmo*^RG/RG^ and heterozygous *kmo*^RG/Δ4^ genotypes, were outcrossed with *kmo*^Δ4/Δ4^, a white-eyed nontransgenic strain with a characterized disruption in *kmo* (40), with G_1_ progeny scored for both fluorescent markers and eye pigmentation to determine the rates of DNA repair proceeding through either the NHEJ or SSA pathways (Fig. 2A). Consistent with SSA-driven elimination of the transgenes, ∼2.7% (16 out of 589) of the progeny of female G_0_ survivors were restored to black eyes (Fig. 2B and C). We observed the NHEJ-driven loss of the DsRED marker alone in 0.7% (4 out of 589) of the progeny of female G_0_ survivors. No SSA-based events and 1 NHEJ were found in the progeny (1 out of 3,276) of male G_0_ survivors. We confirmed that the loss of DsRED in *kmo*^G/Δ4^ mosquitoes was indeed due to imprecise repair at the I-*Sce*I target site resulting in a 4-bp deletion (Figure S2, Supplementary Material). We conclude that it is possible to trigger the complete elimination of transgene sequences, and that SSA-based repair mechanisms can be at least as efficient as NHEJ.

As the timing, level and tissue specificity of I-*Sce*I expression is variable when introduced transiently through plasmid injection, we sought to generate transgenic strains that express I-*Sce*I under the activity of germline-specific *nos* and *beta2-tubulin* (*β2T*), whole-body constitutive *polyubiquitin* (*PUb*), or heat-inducible *heat shock protein 70A* (*Hsp70A*) promoters (37, 41, 42) (Figure S3A, Supplementary Material). Following microinjection to *kmo*^Δ4/Δ4^ embryos, we were able to obtain 1 transgenic mosquito strain each for *Nos-I-SceI* and *PUb-I-SceI*, but none for *β2T-I-SceI* or *Hsp70A-I-SceI*, despite multiple attempts (Figure S3B and Table S3, Supplementary Material). Both *Nos-I-SceI* and *PUb-I-SceI* strains were shown to successfully express *I-SceI* transcripts in embryos at 24 hours postoviposition by RT-PCR analysis (Figure S3C, Supplementary Material), and transgene integration into the mosquito genome was validated by inverse PCR analysis (Table S4, Supplementary Material).

**Fig. 3.**
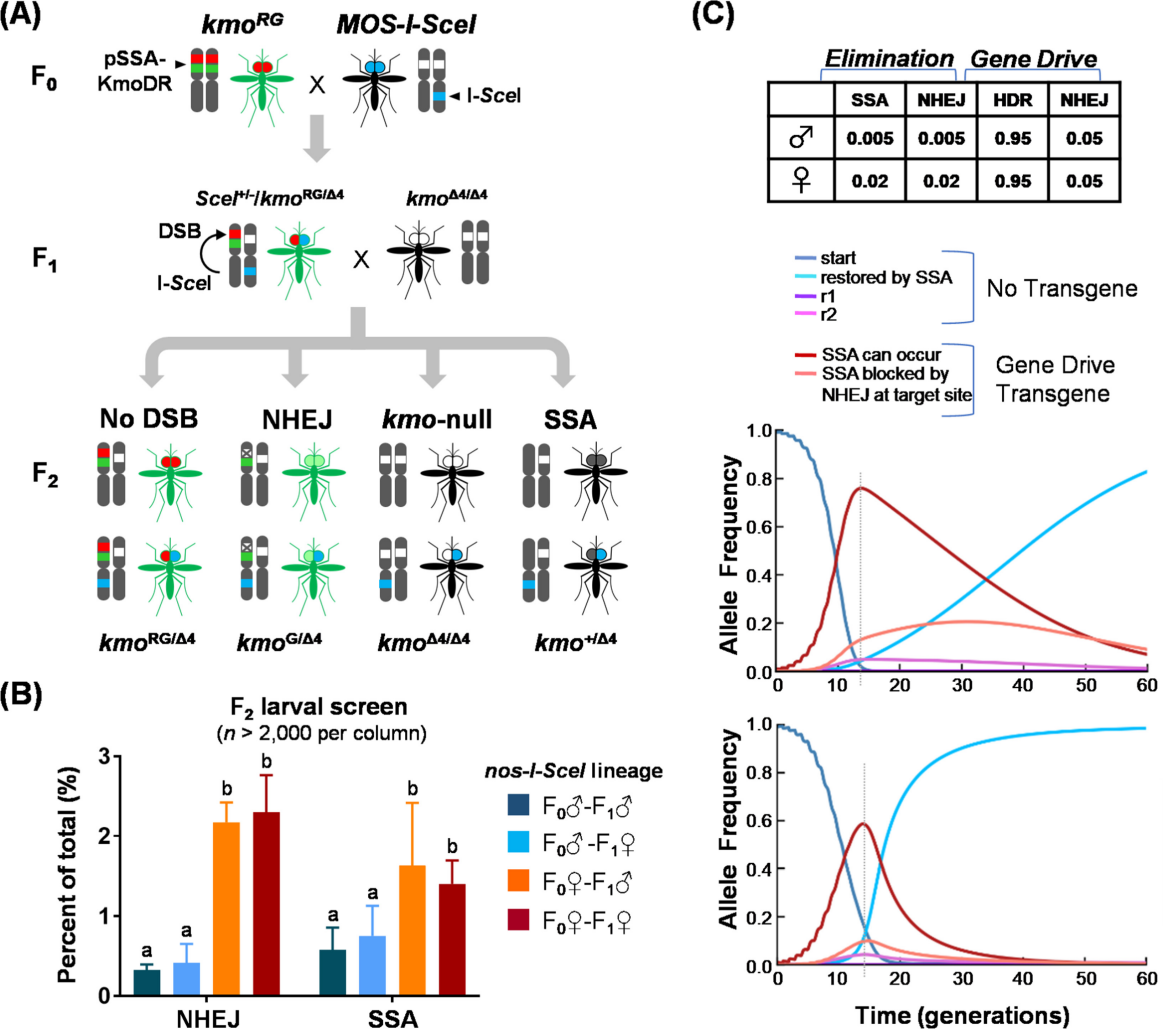
SSA-based transgene elimination in transgenic mosquitoes expressing I-SceI. (A) Schematic representation of crossing scheme used to evaluate the SSA-based transgene elimination in *kmo^RG^* transgenic mosquitoes by reciprocal crossing with *Nos-I-SceI*. (B) Single-generation SSA test using the *Nos-I-SceI* strain (G_12_) as an SSA trigger. F_2_ larvae were scored for marker fluorescence and eye pigmentation to measure the selection frequencies of a DSB repair pathway, either % NHEJ (*kmo*^G/Δ4^/(*kmo*^RG/Δ4^* + kmo*^G/Δ4^* + kmo*^+/Δ4^) or % SSA (*kmo*^+/Δ4^/(*kmo*^RG/Δ4^* + kmo*^G/Δ4^* + kmo*^+/Δ4^). Experimental data were obtained from triplicated tests. Tukey's multiple comparison test was found to be significant (2-way ANOVA, *P* < 0.0002), statistically different groups are marked (a and b). (C) An updated deterministic model of transgene elimination in the context of a homing-based gene drive where the target site is present in a location where functional resistance alleles cannot occur. Parameters for successful (SSA) and failed (NHEJ) transgene elimination are based on (B); gene drive parameters are indicated; all other model parameters are based on (26); (see Supplementary Material). Gene drive scenarios are assuming 5% fitness cost per transgene copy or disrupted host gene (top), or in addition a 100% cost (complete lethality) in females when both copies are disrupted (bottom). Dotted line indicates maximum frequency of the gene drive transgene.

To determine the potential for each strain to initiate SSA-driven transgene elimination, *Nos-I-SceI* or *PUb-I-SceI* mosquitoes were reciprocally crossed with *kmo^RG^* (Fig. 3A). F_1_ individuals that contained both sets of transgenes (*SceI*  ^+/–^/*kmo*^RG/Δ4^) were outcrossed to *kmo*^Δ4/Δ4^ and F_2_ progeny scored for SSA and NHEJ events. In single-generation SSA tests (Table 1 and Fig. 3B; Table S5, Supplementary Material), we observed restoration of the *kmo* gene and complete loss of all transgenes in 0.5%–1% of transgenic progeny when the grandfather (F_0_♂) provided the *Nos-I-SceI* transgene. Likewise, SSA-based repair events constituted 2%–3% of transgenic progeny when the *Nos-I-SceI* cassette was provided by the grandmother (F_0_♀). Interestingly, though the *Nos-I-SceI* cassette was not inherited, the F_0_♀-F_1_ mosquitoes (BFP^–^) were still able to produce DNA repair-associated phenotypes in F_2_ progeny (Table 1), providing evidence that significant numbers of DSBs were induced by the dominant maternal effect of the nuclease. In contrast, no NHEJ or SSA events were recovered when using the *PUb-I-SceI* strain (Table 1), suggesting that expression of I-*Sce*I was insufficient for inducing DSB formation, despite the fact that its transcript was present in embryos (Figure S3C, Supplementary Material). While this result was somewhat unexpected as plasmid-expressed *PUb-I-SceI* did trigger SSA (Fig. 2C), the microinjection procedure into preblastoderm embryos might have allowed the transiently expressed I-*Sce*I enzyme access to the germ cells, enabling DSB repair events to be transmitted to G_1_ progeny, whereas *PUb*-driven *I-SceI* gene expression from the chromosome may be restricted in the germline cells, as *PUb*-driven *EGFP* mRNA was not detectable in the ovarian tissue (37).

**Table 1.** Single-generation tests for SSA-based transgene elimination induced by the I-*Sce*I-expressing trigger strains (G_4_), *Nos-I-SceI* and *PUb-I-SceI*.

			F_2_ Larval screening^a^				
Parental cross (♂30 × ♀100)	Lineage of the SSA trigger^b^	*I-SceI* inherited to F_1_ adults	^c^# Total	# WGR No DSB (*kmo*^RG/Δ4^)	# WG NHEJ (*kmo*^G/Δ4^)	# W *kmo*-null (*kmo*^Δ4/Δ4^)	# Blk SSA (*kmo*^+/Δ4^)
*Nos-I-SceI* x *kmo^RG^*	F_0_♂-F_1_♂	+	7,500	4,122	23 (0.56%)	3,315	40 (0.97%)
		–	7,252	3,609	1 (0.03%)	3,642	0
	F_0_♂-F_1_♀	+	2,588	1,608	9 (0.56%)	957	14 (0.87%)
		–	4,867	2,410	0	2,457	0
	F_0_♀-F_1_♂	+	7,828	4,268	74 (1.73%)	3,380	106 (2.48%)
		–	7,477	3,839	93 (2.42%)	3,528	17 (0.44%)
	F_0_♀-F_1_♀	+	4,676	2,722	36 (1.32%)	1,835	83 (3.05%)
		–	5,749	3,077	10 (0.32%)	2,652	10 (0.32%)
*PUb-I-SceI* x *kmo^RG^*	F_0_♂-F_1_♂	+	494	310	0	184	0
		–	1,211	617	0	594	0
	F_0_♂-F_1_♀	+	1,370	823	0	547	0
		–	2,904	1,473	0	1431	0
	F_0_♀-F_1_♂	+	1,850	1,133	0	717	0
		–	1,302	684	0	618	0
	F_0_♀-F_1_♀	+	1,450	820	0	630	0
		–	1,277	660	0	617	0

a W, white eye; Blk, black eye; G, EGFP; R, DsRED; and B, BFP.

b *nos*-driven germline cell-specific or *PUb*-driven ectopic expression of the HE, I-*Sce*I.

c The *Mariner Mos1*-based transgenic *I-SceI* allele, which is inherited from the parental SSA trigger strain, provides the eye-specific BFP fluorescence.

Mosquitoes scored as *kmo*^G/Δ4^ (NHEJ) and *kmo*^+/Δ4^ (SSA) were confirmed to be heterozygous for the *kmo*-null allele (Figure S4B, Supplementary Material). In addition, mosquitoes scored as *kmo*^G/Δ4^ were associated with a range of melt-curve profiles (Figure S4C, Supplementary Material), indicative of highly diversified indel mutations caused by the NHEJ pathway. Sequencing analysis of F_2_ mosquitoes scored as *kmo*^G/Δ4^ revealed that most indel mutations shifted the *DsRED* gene out-of-frame (Figure S4D, Supplementary Material). However, 1 *kmo*^G/Δ4^ group had a 12-bp in-frame deletion, yet was still scored as phenotypically DsRED-negative. Thus, while we anticipated missing about one-third of NHEJ events (in frame deletions that leave *DsRED* intact), the true number of missed events was likely less than that.

In homing-based gene drive, the conversion of wild-type alleles to transgenics must be a highly efficient process in order to sustain drive (4). However, according to our previous models (26), even low SSA efficiencies of 1%–3%, as shown in this study, should be sufficient to restore a population invaded by a homing-based gene drive transgene to a nontransgenic state. We sought to repeat this modeling effort using these experimentally determined rates of SSA and NHEJ (Fig. 3C), particularly since different rates were observed in male or female founders, a situation we did not explore previously. In each case, gene drive alleles are introduced at a starting frequency of 10% of the total population, and expected allele frequencies for transgene absent [wild type, SSA-restored, gene drive resistant-functional (r1), and gene-drive resistant-nonfunctional (r2)] and transgene containing [SSA-intact and SSA-failed] genotypes, are output each generation. For both homing gene drive into a relatively neutral location (only 5% fitness costs associated with the presence of each copy of the transgene; Fig 3C, top) or into a haplo-sufficient gene critical for female fertility (100% cost in females when 2 copies of the gene drive transgene are present; Fig 3C, bottom), rates of SSA and NHEJ we observed were predicted to be sufficient to effectively restore a nontransgenic state (Fig. 3C, cyan peak) following the initial invasion of the gene drive transgene (Fig. 3C, red peak). Similar to our previous results, the speed at which population-level transgene elimination occurred was inversely proportional to the cost inflicted by the gene drive transgene. Thus, targeting a low-cost genetic locus with self-eliminating gene drive was predicted to restore SSA-driven nontransgenic alleles up to ∼80% after 60 generations, while in the high-cost, female-lethal target the transgene was lost twice as fast (> 80% in 30 generations). In contrast, in the absence of any SSA the transgenes are predicted to remain in their respective populations at high levels in perpetuity in either gene drive approach (Figure S5, Supplementary Material).

**Fig. 4.**
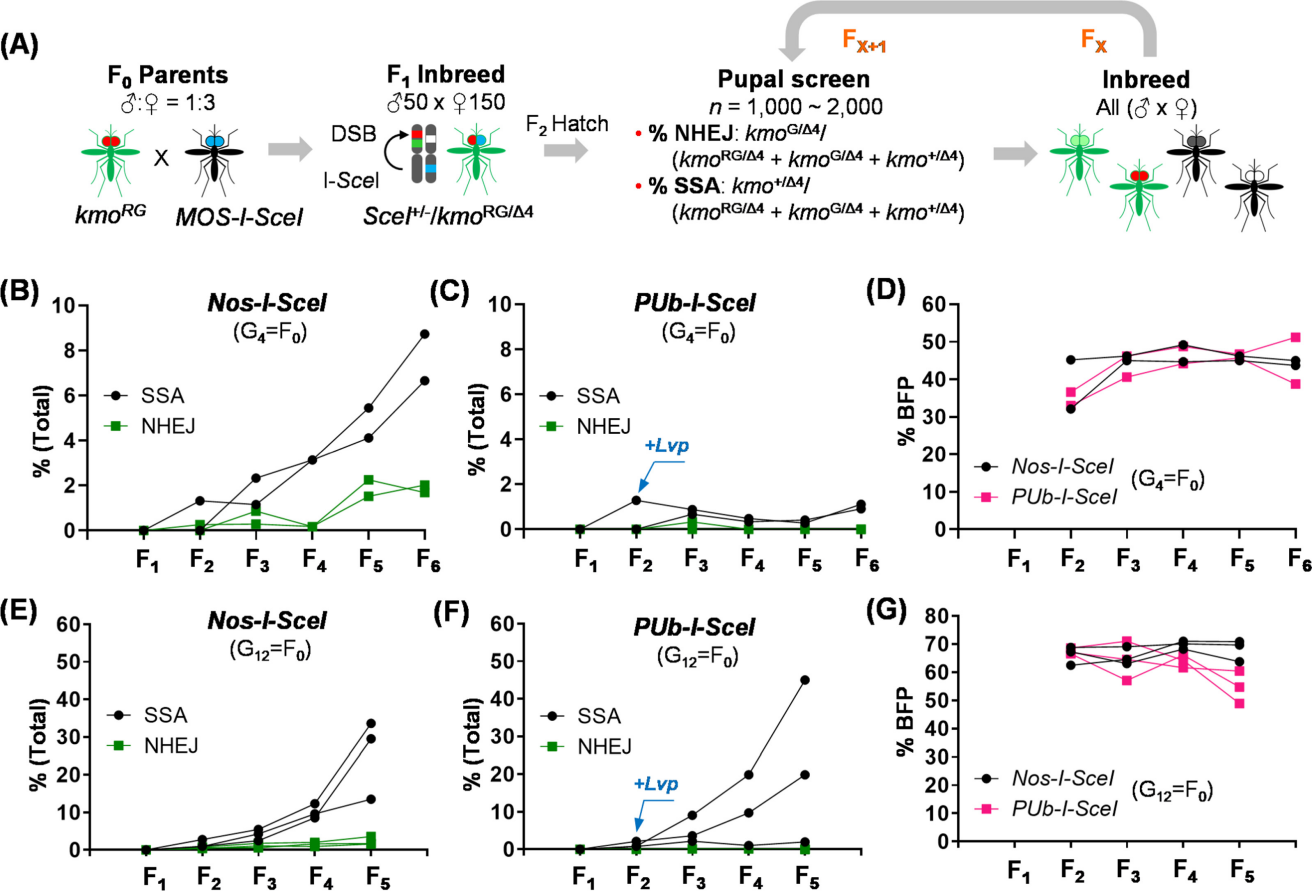
SSA enables progressive transgene elimination from cage populations of *Ae. aegypti*. (A) Schematic representation of the multigeneration SSA test. F_1_ mosquitoes (*SceI*  ^+/–^/*kmo*^RG/Δ4^) from a parental cross (Table 1; Table S5, Supplementary Material) of ♂ *Nos-I-SceI* x ♀ *kmo^RG^* or ♂ *PUb-I-SceI* x ♀ *kmo^RG^* were self-crossed. From F_2_ screening, DSB repair-associated marker phenotypes, % NHEJ (*kmo*^G/Δ4^/(*kmo*^RG/Δ4^* + kmo*^G/Δ4^* + kmo*^+/Δ4^), and % SSA (*kmo*^+/Δ4^/(*kmo*^RG/Δ4^* + kmo*^G/Δ4^* + kmo*^+/Δ4^), were scored for 1,000–2,000 pupae, and all of them were emerged in the same cage for the next generation, up to F_5_ or F_6_. (B)–(G) The multigeneration SSA tests using the SSA trigger strains at G_4_ (B)–(G) or G_12_ (E)–(G). Percentages of DNA repair pathway-dependent phenotypes were scored for the SSA trigger, *Nos-I-SceI* (B) and (E) or *PUb-I-SceI* (C) and (F), from the F_2_ generation. For the control experiment with *PUb-I-SceI* mosquitoes, black-eyed *Lvp* mosquitoes were added by equal numbers of mosquitoes identified as *kmo*^+/Δ4^ in *Nos-I-SceI* at the F_2_ generation [(C) and (F), blue arrows]. Frequencies of *Nos-I-SceI* or *PUb-I-SceI* were scored by the BFP^+^ percentages out of total pupae (D) and (G). Graphs represent data from 2 biological replicates for G_4_ (Table S6, Supplementary Material) and 3 biological replicates for G_12_ (Table S7, Supplementary Material).

As a preliminary test of these models, we allowed *kmo^RG^* mosquitoes to interbreed with *Nos-I-SceI* or *PUb-I-SceI* mosquitoes in order to observe if the SSA-based rescue system would be capable of removing transgenes from the *kmo^RG^* mosquito population over multiple generations (Fig. 4). To do this, we self-crossed F_1_ mosquitoes heterozygous for each transgene (*SceI*  ^+/–^/*kmo*^RG/Δ4^) inherited from an F_0_ cross between ♂ *Nos-I-SceI* or *PUb-I-SceI* and ♀ *kmo^RG^* mosquitoes (Fig. 4A and Table 1; Table S5, Supplementary Material). For each generation starting from F_2_, we hatched about 1,000–2,000 embryos and scored all pupae for eye pigmentation and fluorescence to determine DSB repair events, with all individuals placed into a large cage to establish the next generation (Fig. 4A). The cages were kept in complete darkness for 1 week to reduce any potential competitive advantage provided by those individuals with wild-type eye pigmentation during mating.

For the *Nos-I-SceI* x *kmo^RG^* experiment initiated at the G_4_ generation with respect to the establishment of the Nos-I-SceI strain, 5 F_2_ individuals with wild-type black eyes (Blk) were identified from 765 *kmo*^RG/Δ4^ mosquitoes (0.7%), with the number of individuals with the restored phenotypes increasing by 10-fold when the experiment was concluded at F_6_ (Fig. 4B and Table S6, Supplementary Material). To determine whether this increase was due to new SSA events each generation or to a selective advantage provided by the restoration of *kmo*, we performed a parallel control experiment with *PUb-I-SceI* mosquitoes. As no SSA events were detected (as expected), this population was supplemented with the addition of 5 wild-type individuals at the F_2_ generation. No change in wild-type *kmo* allele frequency was observed in the *PUb-I-SceI* x *kmo^RG^* experiment (Fig. 4C; Table S6, Supplementary Material), indicating the increase in wild-type, nontransgenic alleles in the *nos-I-SceI* experiment appeared to be due to SSA-based repair of I-*Sce*I-induced DSBs and not to any competitive advantage of the wild-type over their white-eyed relatives. However, when this multigeneration SSA test was repeated using the SSA trigger strains at the G_12_ generation, the frequencies of black-eyed individuals in the spike-in control cage populations were more variable (2–10-fold; Fig. 4E and F; Table S7, Supplementary Material). This surge of wild-type mosquitoes may be related to some early-emerging wild-type mosquitoes that dominate initial mating events in a highly closed environment of the caged population, as the mating competition tests did not show any *kmo*-associated advantage of wild-type mosquitoes for producing progeny under the experimental conditions used (Figure S6, Supplementary Material). Thus, while at this point we cannot separate the precise contributions of SSA/selection in increasing the frequency of the wild-type trait, these results confirm that SSA can generate a sufficient number of wild-type individuals to allow selection to act.

Interestingly, the frequency of restored wild-type individuals increased much faster in the G_12_ experiment (30%–40% at F_5_) as compared to the G_4_ experiment (less than 10% at F_6_). One potential explanation for this is due to greater exposure to the I-*Sce*I nuclease [average allele frequency was 44.2% in experiment 1 (G_4_), and 67% in experiment 2 (G_12_; Fig. 4D and G)]. This suggests that with complete linkage (allele frequency 100%) with the *I-SceI* transgene if encoded at the target locus itself, the frequency of DSB induction, and hence repair could likely be even higher than the current split-type system, where the nuclease and the target transgene are engineered in independent strains at different genomic loci and trigger DSBs only when both components are transmitted together. Compared to SSA-associated alleles (*kmo*^+/Δ4^), NHEJ-driven indels in *DsRED* (*kmo*^G/Δ4^ and *kmo*^G/+^) were shown to occur at lower frequencies (average ∼1%; Fig. 4B and E; Tables S6 and S7, Supplementary Material) and while these events were also identified in the *kmo^RG^* group every generation (*kmo*^G/RG^), they did not increase over time (Figure S7, Supplementary Material). Taken together, we conclude that *nos*-driven I-*Sce*I expression can reliably induce the removal of transgene sequences, and the resulting SSA-repair can faithfully restore the disrupted gene in *Ae. aegypti*.

## Discussion

While technical improvements in gene drive transgenes continue to accumulate in laboratory-based experiments, their impacts on local environments and the ultimate behavior of these technologies in field-based settings remains unknown. Despite great promise in the fight against malaria and other vector-borne diseases, these uncertainties are of concern to relevant stakeholders, with evidence demonstrating that such concerns are somewhat eased by making gene drive transgenes reversable or limited (19–21). Self-limiting and confinable gene drive systems (i.e. split drive and daisy-chain drive) and CRISPR-gene drive brake systems (i.e. CATCHA, ERACR, eCHACR, and anti-CRISPR protein) have been evaluated in laboratory settings to halt the gene drive process, but not to erase the transgene itself from the test field (10, 22–25).

**Fig. 5.**
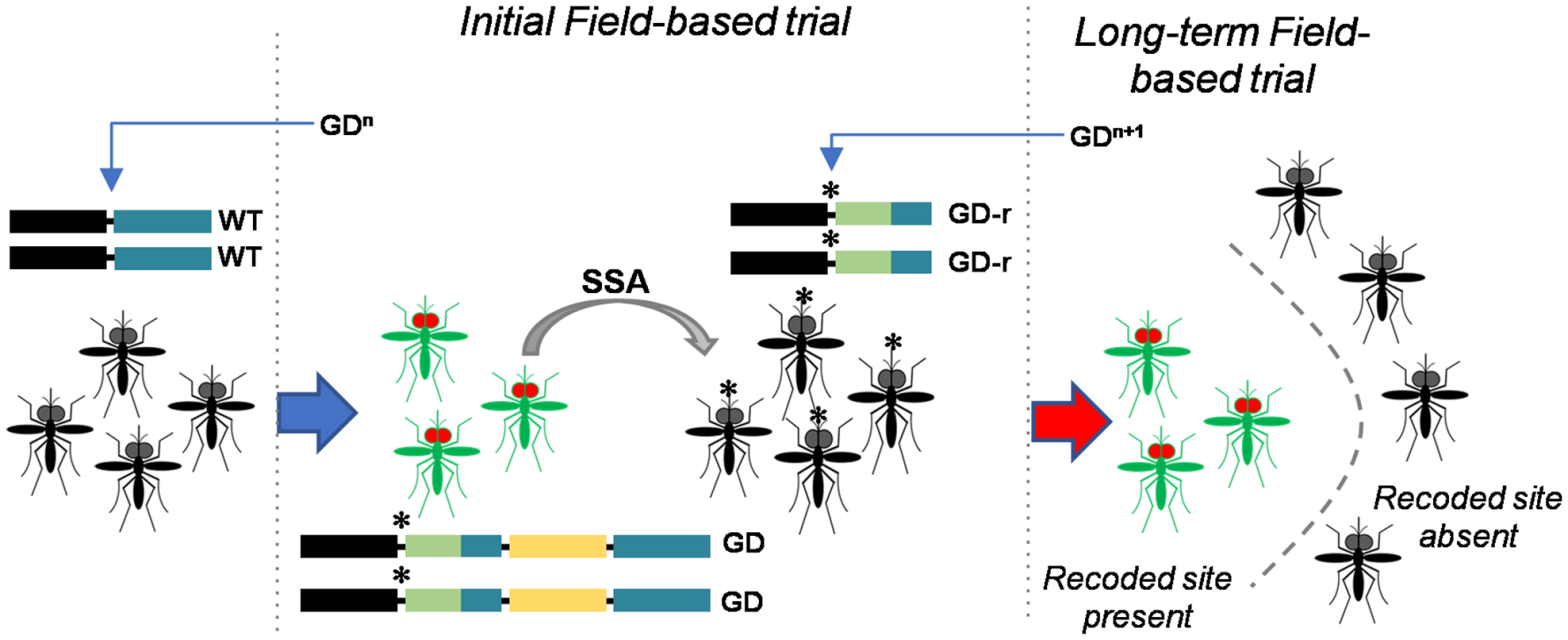
A 2-step process for field-based evaluation of gene drive transgenes. In step 1, risk assessment, engagement activities, and regulatory decisions for an initial trial would be based on a self-eliminating gene drive approach (GD^n^), at the end of which the target population would return to a nontransgenic state and be resistant to the gene drive transgene used (GD-r). The outputs from this limited trial would inform risk assessment, engagement, and regulatory actions regarding proceeding to step 2 using a second gene drive transgene (GD^n+1^), where SSA-based limitations may no longer be needed. Importantly, GD^n+1^ would not be able to spread to any area that did not receive the first GD, since the recoded target site would not be present. For all practical purposes, GD^n^ and GD^n+1^ would be highly similar, potentially differing only in the gRNAs used to generate the DSB (arrows) and the corresponding recoded HA (green).

Our current work suggests a potential role for an SSA-based rescue strategy in removing transgenic gene cassettes in the targeted population by both removing the effector gene, while simultaneously restoring a wild-type allele from the gene drive allele. A single component system consisting of both a homing-based gene drive and an SSA-based self-elimination mechanism at a single locus is predicted to allow the temporary invasion of a gene drive transgene (allowing potential field testing), with SSA-triggered reversion to wild-type occurring with no need for remediation such as the inundated release of wild-type strains (26). This suggests an alternative in how gene drive field trials could be conceptualized, from a single trial format where uncertainty is highest and removal/reversal of the gene drive transgene may not be possible, to a 2-step format (Fig. 5). Here, an initial trial is performed with the gene drive transgene bounded by the SSA-elimination mechanism. Whether the trial concludes as planned, is interrupted, or is stopped prematurely, the population reverts back to a nontransgenic state, eliminating the engineered transgenes and leaving just silent or neutral variants expected to mimic naturally occurring variants. While the recoding of the homing target site prevents a reuse of an identical gene drive transgene, it also creates a novel private allele (43, 44) that can be exploited by a second gene drive used in the next step (Fig. 5). Given the ease of generating new gRNAs for CRISPR/Cas9 systems, this is likely to be trivial. Since the recoded target region is predefined, both GD^n^ and GD^n+1^ could be prevalidated in laboratory trials at the same time, while the dependence on the recoded allele would put strict spatial limitations on the spread of the gene drive transgene during the long-term phase.

We note, however, that this technology is not limited to CRISPR homing drives, and would allow any transgene to be degradable by itself. Thus, SSA-based transgenes could also be incorporated into almost all transgenesis-based genetic control approaches, including split drive (10), daisy-chain drive (22), ClvR (45), Medea (46), and toxin-antidote (47) that utilizes composite interactions of multiple transgenes, potentially shortening the lifespan of 1 or more components. In addition, the recoded allele generated by SSA might be considered an end in and of itself. For example, host factors required by malaria parasites or arboviruses could be potentially recoded in a manner that preserves their cellular functions but prevents their exploitation by these pathogens (Figure S8A, Supplementary Material). Thus, population replacement approaches might be possible that do not rely on the long-term presence of engineered transgenes. Similarly, SSA-based elimination could be designed to remove only critical elements of the gene drive transgene, while leaving associated antipathogen cargo genes in place (Figure S8B, Supplementary Material). Finally, recoding of the gene drive target sites is needed not just to prevent reinvasion of the gene drive transgene, but to restrict competition between the engineered direct repeat and the preferred HA during the process of homing (Figure S8C, Supplementary Material), which could otherwise short-circuit the gene drive process and prematurely restrict the spread of the transgene. An analysis by Lopez del Amo et al. (48) indicated that even disruptions in homology of as few as 20 bps from each end at the break site substantially reduce homing rates, indicating that this unwanted competition could be prevented.

While the rates of successful transgene removal via SSA and the rates of competing NHEJ we observed varied from 0.5% to 3%, nonetheless these values are anticipated to be sufficient to counteract homing-based gene drive approaches. In fact, the SSA rate we observe may be relatively close to optimal as substantially higher SSA activity may destabilize the gene drive transgene prematurely and lead to the establishment of SSA-resistant transgenes (26). Given this, we anticipate several parameters that could be optimized for better efficiency of transgene removal (i.e. the choice between SSA and NHEJ outcomes), such as the length and spacing of the DR, the type, timing, and expression level of the nuclease used, and the number of DSBs induced and their proximity to the DRs. For example, the efficiency of SSA-based repair is dependent on the lengths of DRs, and it is also preferred when DSBs are closest to DRs (49, 50). Our results revealed that ∼0.7 kb of DRs were able to delete ∼3.7 kb of the intervening genes in a heritable manner in *Ae. aegypti*, when the I-*Sce*I-digested DSB was induced at 327 bp away from 1 of the DR sequences. Determining the optimal length of DR and its distance to the DSB according to the gene drive cargo sizes would be required in the application of this technology. While here I-*Sce*I was successfully engineered to induce germline-specific DSBs to activate SSA, other HEs such as I-*Ani*I and I-*Cre*I have also been to catalyze DSBs in the *Ae. aegypti* genome (35, 51); alternatively, independent sgRNAs could be included to those used to catalyze the process of gene drive. In addition, tissue- or cell-specific expression of the endonuclease may be critical for optimal SSA-based repair.

In conclusion, our study demonstrates that the core molecular elements of SSA, 2 flanking DRs (*kmo*), and a cargo-specific DSB by I-*Sce*I, are effective for erasing 2 transgenes (*DsRED* and *EGFP*) from a GM mosquito strain. More interestingly, these SSA motifs were able to restore the transgene-inserted *kmo* allele flawlessly, and thereby, rescue the wild-type phenotypic trait. This seamless recovery of the targeted gene persistently occurred across multiple generations by *nos*-driven germline-specific SSA activation. As SSA-based repair is shared by diverse organisms; *Drosophila melanogaster* (52), *Ae. aegypti* (35), *Saccharomyces cerevisiae* (34), *Arabidopsis thaliana* (53), *Caenorhabditis elegans* (54), and mammalian cells (33), this rescue technology should be amenable for potentially broad applications with a species-specific, spatial-temporal activation control.

## Materials and Methods

### Mosquito rearing

The *Ae. aegypti* Liverpool wild-type strain (*Lvp*), the TALEN-generated *kmo*-null mutant strain (*kmo*^Δ4/Δ4^) (40), and all transgenic strains were maintained at 27°C and 70% (±10%) relative humidity, with a day/night cycle of 14 hours light and 10 hours dark. Larvae were fed on ground dry fish food (Tetra), and adult mosquitoes were fed on 10% sucrose solution. The mated females were fed on defibrinated sheep blood (Colorado Serum Company) using the artificial membrane feeder.

### Subcloning

To generate pSSA-KmoDR0.7, the donor DNA for *kmo^RG^*, 3 plasmids (pGSP1-KmoHA1-DR0.7, pGSP2.3-DsRED-SV40, and pGSP3.8C-EGFP-KmoHA2) were modified from the synthesized plasmid templates (GenScript) and assembled by Golden Gate Assembly (NEB). pGSP1-KmoHA1-DR0.7 contained *kmo* exon4/5 (HA1) and *kmo* exon2/3 (HA2), direct repeat [DR]). pGSP2.3-DsRED-SV40 encoded *3xP3-DsRED-SV40*, in which the HE I-*Sce*I recognition site (5“-TAGGGATAACAGGGTAAT-3”) was engineered in-frame next to the ATG translation start codon of DsRED. pGSP3.8C-EGFP-KmoHA2 included the *PUb-EGFP-SV40* and *kmo* exon2/3 (HA2, DR). For the donor DNA for *kmo^EGFP^*, Golden Gate Assembly using pGSP1-KmoHA1, pGSP2-REDh-SV40, and pGSP3.8C-EGFP-KmoHA2 generated pBR-KmoEx4. pGSP1-KmoHA1 was made by replacing the *kmo* exons 2-to-5 sequence in pGSP1-KmoHA1-DR0.7 with the KpnI-AgeI fragment of *kmo* exon4/5. pGSP2-REDh-SV40 was modified from pGSP2.3-DsRED-SV40 by removing the AscI and SbfI fragment of the *3xP3* promoter and the 5'-half of *DsRED* containing the I-*Sce*I site. Sequential blunting and ligation of both enzyme-cut ends (AscI and SbfI) created the sgRNA-HybRED site that is unique to REDh.

To generate I-SceI-expressing transgenic strains, *Mos1*-based plasmid constructs were assembled with I-*Sce*I under the control of several promoters known to function in *Ae. aegypti*; *nos* (41, 55), *β2-tublin* (56), *PUb* (37, 42), and *Hsp70A* (37, 42). In total, 2 steps were taken for assembling the donor plasmid constructs. First, the MluI-BamHI fragment of *nos* (∼1.56 kb) or *β2-tublin* (∼1.0 kb) promoter, the BamHI-SalI fragment of the *I-SceI* coding region (∼0.85 kb), and the NotI-EcoRI fragment of *nos* (∼0.5 kb) or *β2-tublin* (∼0.2 kb) 3“-UTR were obtained by PCR amplifications using primer sets providing the corresponding enzyme sites (Table S2, Supplementary Material) and sequentially assembled into a universal insect plasmid backbone pSLfa-PUb-mcs (Addgene #52908) to generate pSLfa-Nos-I-SceI or pSLfa-β2T-I-SceI. For pSLfa-PUb-I-SceI, the I-*Sce*I coding sequence was ligated to BamHI and SalI sites in pSLfa-PUb-mcs. For pSLfa-Hsp70A-I-SceI, the MluI-NcoI fragment of *Hsp70A* promoter (∼1.5 kb) was replaced for *PUb* promoter (∼1.4 kb) in pSLfa-PUb-I-SceI. Second, the whole DNA piece of Promoter-I-*Sce*I-3'-UTR was taken out from the individual pSLfa-based plasmid construct and inserted to MluI and EcoRI sites in pM2-3xP3-BFP, a *Mariner Mos1*-based plasmid backbone. Complete sequences of plasmid constructs (pBR-KmoEx4, pSSA-KmoDR0.7, and pM2-3xP3-BFP-Nos-I-SceI) used to generate transgenic mosquito lines are deposited as Supplementary Files (S1–S3).

### Generation of *kmo^EGFP^* and *kmo^RG^* strains

Site-specific integrations at the *Ae. aegypti kmo* site were obtained by microinjection into preblastoderm embryos as previously described (57–59). For the *kmo^EGFP^* strain, the injection mix included 0.4 µg/µl of CRISPR/Cas9 enzyme (PNA Bio), 0.1 µg/µl of sgRNA-KmoEx4, and 0.3 µg/µl of donor plasmid pBR-KmoEx4 was microinjected to the *Lvp* wild-type embryos. The G_2_  *kmo^EGFP^* strain was utilized as a recipient for a second round of microinjections using sgRNA-HybRED, Cas9, and pSSA-KmoDR0.7 (same concentrations as above) to generate the *kmo^RG^* strain. Chromosomal integration of the transgenes at the *kmo* locus was confirmed by PCR analysis using genomic DNAs purified from a single G_2_ individual larva as the template and a primer set that is specific to the transgene or *kmo* (Fig. 1D; Table S2, Supplementary Material). PCR was performed using the Phusion High-Fidelity DNA polymerase (NEB) for 35 cycles: 95°C for 30 seconds, 58°C for 30 seconds, and 72°C for 2 minutes.

### Generation of *MOS-I-SceI* strains

To generate transgenic strains expressing I-SceI, each donor plasmid (0.5 µg/µl), pMOS-3xP3-BFP-Nos-I-SceI, pMOS-3xP3-BFP-β2T-I-SceI, pMOS-3xP3-BFP-PUb-I-SceI, or pMOS-3xP3-BFP-Hsp70A-I-SceI, was microinjected into preblastoderm embryos of the *kmo*-null (*kmo*^Δ4/Δ4^) strain (40), along with the *Mos1* helper plasmid (0.2 µg/µl), pKhsp82M (60). For BFP-positive transgenic mosquitoes, transposon–chromosome junction sequences were identified by inverse PCR using Sau3AI-digested genomic DNA and primers indicated in Figure S3A and Table S2 (Supplementary Material). For the evaluation of I-*Sce*I transcripts, total RNA was extracted from 200 embryos at 24 hours after oviposition using the Trizol reagent (Invitrogen). First-strand cDNAs were synthesized from 1 µg of total RNAs using the SuperScript IV VILO Reverse Transcription Kit (Life Technologies). To amplify the transcript-derived cDNA of I-*Sce*I, PCR was performed using the Q5 High-Fidelity DNA polymerase (NEB) and I-*Sce*I gene-specific primers (Table S2, Supplementary Material) with 35 cycles; 95°C for 30 seconds, 60°C for 30 seconds, and 72°C for 1 minute.

### Single-generation SSA tests

For experiments using a plasmid-based source of I-*Sce*I, 0.5 µg/µl of pSLfa-PUb-SceI (61) was microinjected into *kmo^RG^* recipient embryos obtained from parental self-crossing between heterozygous mosquitoes. Since a mixture of transgenic (75%) and nontransgenic (25%) offspring were expected from this cross, only EGFP^+^/DsRED^+^ survivors were further outcrossed to the *kmo*^Δ4^ strain. G_1_ larvae were scored for either white or black eyes under visible light, and for eye-specific DsRED or whole body EGFP fluorescence using the appropriate excitation/emission filters. For experiments using the germline-based I-*Sce*I transgenic strains, homozygous *kmo^RG^* mosquitoes were reciprocally crossed with the *Nos-I-SceI* or *PUb-I-SceI* mosquitoes in a cage of 30 males and 100 females at G_4_ or 20 males and 50 females in triplicate at G_12_. A total of 50 male or female F_1_ progenies (Kmo^–^/EGFP^+^/DsRED^+^/BFP^+^; *SceI*  ^+/–^/*kmo*^RG/Δ4^) were outcrossed with the *kmo*^Δ4^ strain in a ♂: ♀ ratio of 1:3. Female mosquitoes were blood-fed 3 times, and all subsequent embryos were hatched for F_2_ larval screening.

### Multigeneration SSA test

Thirty Nos-I-SceI or PUb-I-SceI males were crossed with 100 kmo*^RG^* females, to establish each F_0_ cage. Only individuals scored positive for all marker phenotypes (Kmo^–^/EGFP^+^/DsRED^+^/BFP^+^; *SceI*  ^+/–^/*kmo*^RG/Δ4^) were selected for the F_1_ cage of 50 males and 150 females. For each generation from F_2_, approximately 1,000–2,000 embryos were hatched for phenotypic examinations. In the control cage with *PUb-I-SceI*, we added the same numbers of wild-type individuals as identified in Nos-I-SceI at the F_2_ generation. Male or female pupae were first separated based on eye pigmentation [black-eyed (*kmo*-haplosufficient) or white-eyed (*kmo*-knockout)]. Pupae were next screened for EGFP (G) and DsRED (R) fluorescence to identify *kmo*^Δ4/+^, *kmo*^RG/+^, *kmo*^G/+^, *kmo*^Δ4/Δ4^, *kmo*^RG/Δ4^, and *kmo*^G/Δ4^. All groups were then subsequently screened for BFP to track the frequency of the I-SceI transgene in each phenotypic group. Once scored, all pupae regardless of phenotype were placed in cages for the next generation. Both male and female pupae were kept in complete darkness for 1 week, when the adults emerged and completed mating, to reduce any competitive advantage provided by those individuals with wild-type eye pigmentation during mating, after which they were returned to the normal day/night light cycle.

### Mating competition assay

To determine eye color-dependent mating efficiency, we set up 3 replicates of mating enclosures (46 oz. food cups), each of which contains 25 wild-type (*kmo*^+/+^) males, 25 *kmo*-null (*kmo*^Δ4/Δ4^) males, and 50 virgin *kmo*-null (*kmo*^Δ4/Δ4^) females at 28°C, 85% humidity, and a light intensity of ∼12 lux. To give all males an equal opportunity to mate, the 2 male groups were put into the enclosure prior to introduction of females. The females were individually oviposited in 3 days postblood-feeding by the EAgaL plate method (62), and the eggs per female were hatched for independent larval scoring of eye pigmentation.

## Supplementary Material

pgac037_Supplemental_File

## Data Availability

All data are presented within the manuscript or are available in the Supplementary Material.
