# Supplementary material for: Engineering a self-eliminating transgene in the yellow fever mosquito, Aedes aegypti
Source: PNAS Nexus. 2022 Mar 30;1(2):pgac037. doi: 10.1093/pnasnexus/pgac037 (PMC9802104; doi:10.1093/pnasnexus/pgac037)
Supplement: pgac037_Supplemental_File [file pgac037_supplemental_file.docx]

**Supplementary Information for**

**Engineering a self-eliminating transgene in the yellow fever mosquito *Aedes aegypti***

Keun Chae, Chanell Dawson, Collin Valentin, Bryan Contreras, Josef Zapletal, Kevin M. Myles, and Zach N. Adelman*

Department of Entomology, Texas A&M University, College Station, TX 77843, USA

*Correspondence to Zach N. Adelman

Email: zachadel@tamu.edu

**Supplemental Text**

Model Structure

The basic model structure follows as described in (26).

The population model was broken down into juvenile and adults. Upon initialization, the initial adults mated and the juvenile population was seeded. Adults continue to seed juveniles according to the number of adults surviving from their cohort at each time step, until less than 1% of the adult cohort was surviving. Juveniles developed into adults following their development period (η), at which time they start a new cohort and mate to begin seeding the subsequent generations.

The model returned total counts of juveniles and adults by genotype, from which the proportions of each allele were calculated. The equation generation, model execution, and plotting of results were done using Python 3.9.1. Initial model parameters and gene drive mechanism parameters are provided below:

**Variable definitions**

| **Variable** | **Description** | **Value** |
| --- | --- | --- |
| λ | Female reproduction rate (per day) | 7 |
| σ | Proportion of female offspring | 0.5 |
| $c_{i}$ | Fitness cost of genotype *i* | 0.05 per transgenic or nonfunctional allele (*g, s, r*) |
| $\mu_{A}$ | Adult mortality rate (per day) | 0.1 |
| $\mu_{J}$ | Juvenile mortality rate (per day) | 0.03 |
| η | Development time (in days) | 12 |

**Gene drive mechanism parameters**

| Parameter | Description | Value |
| --- | --- | --- |
| *q* | Probability of Cas9 cut to cause DSB | 0.95 |
| *p* | Probability of successful HDR | 0.95 |
| *δ* | Probability of functional allele formation through NHEJ | 0 |
| *1-δ* | Probability of non-functional allele formation through NHEJ | 0.05 |

Role of Fitness Cost

Fitness cost (*c)* associated with transgenic and non-function alleles were incorporated into the model by impacting the reproduction rates and mortality rates of affected genotypes. The reproduction rates ($\lambda$), or the number of offspring of each genotype created at each model time step, were calculated such that:

$$\lambda_{i}=\lambda(1-c_{i})$$

Fitness cost also increased the daily mortality rates for adults such that adult mortality rates were calculated as:

$$\mu_{A_{i}}= \mu_{A}(1+c_{i})$$

Likewise, juvenile mortality rates were calculated as:

$$\mu_{J_{i}}= \mu_{J}(1+c_{i})$$

Reproduction Rates

The number of offspring produced in each time step was dependent on the number of males and females present in each adult cohort. We assume that females only mate once with the males in their cohort. Hence the proportion of males at the time of mating was used to determine the generation of subsequent genotypes.

Equation Generation

The equations determining the number of offspring of each genotype follow the same logic as those in (26). To make the models open-source, Python was used instead of MATLAB.

Model Execution

The model package consists of six total components, of which five are required to run the model and output the results. The following components must be located within the same folder in order to execute the model without errors:

- *CRISPR1-1_main.py*
- *CRISPR1_1_model.py*
- *CRISPR1_1_Equations.py*
- *CRISPR1-1 Input Parameters.xlsx*
- *Allele_Plots.py*

Model parameters can be modified within the *CRISPR1-1 Input Parameters.xlsx* Excel file. The file must be saved after any changes to be read in and executed properly by Python. The model can then be executed by running the *CRISPR1-1_main.py* module. The primary output will consist of allele plots for all the scenarios entered in the *CRISPR1-1 Input Parameters.xlsx* Excel file. The quantitative results from which these plots were generated will be stored in the file *CRISPR1_1 Results.xlsx.* These results are overwritten each time the model is run and store the proportion of each allele and population size at each time step for all scenarios.

The *CRISPR1_1_Equation_Generation.py* module is not required to execute the model, but provides the framework from which the equations within the *CRISPR1_1_Equations.py* were generated.

These files can be found at: *https://github.com/jozinzapletal/Python-CRISPR1-1-SEM-gene-drive*

**Supplemental Figure legends**

Fig. S1. The sgRNAs used for the development of *kmo^EGFP^* and *kmo^RG^* strains. (*A*) The sgRNA-KmoEx4 was designed to target the 4^th^ exon of the *Ae. aegypti* *kmo* gene locus, which is the landing site for HDR-mediated knock-in to generate *kmo^EGFP^* strain (see Fig. 1*B*). PCR primers used for High Resolution Melting Analysis (HRMA) KmoEx4-F and KmoEx4-R are shown (horizontal arrows). The indel-associated melting curve variants are shown in different colors, compared to the LVP controls (gray). (*B*) The sgRNA-HybRED was designed to recognize *RED*_1/2_ in pBR-KmoEx4 created by blunted-end fusion of AscI and SbfI cuts. This allows for the HDR-mediated integration of the donor DNA, pSSA-KmoDR, to generate the *kmo^RG^* strain (Fig. 1*B*). HRMA using a PCR primer pair of KmoEx4-F and DsRED-3R (horizontal arrows) showed efficient activity of sgRNA-HybRED to result in DSB-induced indel mutations in *kmo^EGFP^* strain. The indel-associated melting curve variants are shown in different colors compared to the untreated controls (gray).

Fig. S2. Verification of the indel mutations following microinjection of a plasmid expressing I-*Sce*I to *kmo^RG^* embryos. (*A*) Schematic representation of the transgene structure in the *kmo^RG^* strain; direct repeats (DR) are indicated. (*B*) HRMA of the I-*Sce*I site in *DsRED* for G_1_ mosquitoes scored as *kmo*^RG/Δ4^ or *kmo*^G/Δ4^ using primers DmHsp70-F and RED-3R (horizontal arrows, Fig. S2*A*). The indel-associated melting curve variants are shown in different colors. (*C*) Sequencing analysis revealed a 4 bp deletion mutation in G_1_ mosquitoes scored as *kmo*^G/Δ4^ (Fig. S2*B*). The ATG in bold letters is the translation start codon of *DsRED* gene and the I-*Sce*I recognition site is underlined.

Fig. S3. *Aedes aegypti* transgenic mosquitoes as SSA triggers. (*A*) Schematic representation of *Mariner Mos1*-based plasmid DNA constructs expressing *I-SceI* under the control of various promoters: *nos* and *β2-tublin* for female- and male-specific germline cells, respectively, and *PUb* and *Hsp70A* for ectopic and heat-inducible gene expression, respectively. *Mos1* IRR, *Mos1* inverse repeat right; *Mos1* IRL, *Mos1* inverse repeat left. (*B*) SSA trigger strains expressing BFP marker in their eyes in both adults and 4^th^ instar larvae. (*C*) RT-PCR analysis for *I-SceI* gene expression in SSA trigger strains using primers SceI-F and SceI-R for *Nos* or *PUb*-driven *I-SceI* transcripts or the S7 primer pair for 40S ribosomal protein gene (*RPS7*) as the RNA control. The *kmo*-null (*kmo*^Δ4/Δ4^) strain was included as the control of no *I-SceI* transgene. To control for the potential presence of genomic DNA, the same analysis was performed on the side in the absence of the reverse transcriptase (RT-).

Fig. S4. Verification of DSB repair-associated phenotypes following reciprocal crosses between *kmo^RG^* and the *Nos-I-SceI* strain. (*A*) Schematic representation of the transgene structure in the *kmo^RG^* strain, with primers pairs indicatd used in HRMA indicated. (*B*) HRMA utilizing the PCR primer pair of KMR1 and KMF2 identified *kmo*^Δ4^ allele variations in F_2_ mosquitoes. The indel-associated melting curve variants are shown in different colors. (*C*) HRMA utilizing the PCR primer pair of DmHsp70-F and RED-5Ra identified sequence variations generated by I-*Sce*I-induced DSBs in F_2_ mosquitoes scored as *kmo*^RG/Δ4^ or *kmo*^G/Δ4^. The indel-associated melting curve variants are shown in different colors. (*D*) Sequencing analysis showed various indel mutations resulted from a I-*Sce*I-induced DSB in F_2_ mosquitoes scored as *kmo*^G/Δ4^. The sequence ID has the same color as that of melting curve variants shown in (C). The ATG in bold letters is the translation start codon of *DsRED* and the I-*Sce*I recognition site is underlined. Red-colored letters indicate the newly inserted nucleotides and green-colored letters indicate nucleotide changes.

Fig. S5. Deterministic model of transgene elimination in the context of a homing-based gene drive where the target site is present in a location where functional resistance alleles cannot occur. Parameters for successful (SSA) and failed (NHEJ) transgene elimination are set to zero, otherwise all parameters are the same as those in Fig. 3*C*. Gene drive scenarios assume a 5% fitness cost per transgene copy (*A*) or 100% cost (complete lethality) in females when both copies are disrupted (*B*).

Fig. S6. The eye pigmentation-dependent mating competition assay. (*A*) Illustration of the workflow for the mating competition assay between wild-type and *kmo*-null mosquitoes. (*B*) Phenotypes of F_1_ offspring obtained from the mating competition test. Each bar represents the percentage of females that produced progeny with black eyes (Blk) or white eyes (W), or a combination of both types of progenies (Blk+W). Results were obtained from three biological replicates. Tukey’s multiple comparisons test (1-way ANOVA): P<0.05. (*C*) Fertility of individual F_0_ females. Each dot represents the number of larvae produced from a single female. Blk, black-eyed progeny; W, white-eyed progeny. Unpaired t test: P=0.17.

Fig. S7. The emergence of SSA-resistant alleles in a cage population of *kmo^R^*^G/Δ4^ mosquitoes during the multi-generation SSA test. (*A*) Schematic representation of the transgene structure in the *kmo^RG^* strain. DmHsp70-F and RED-5Ra (horizontal arrows) are PCR primers to identify sequence variations generated by I-*Sce*I-induced DSBs. (*B* to *E*) HRMA for I-*Sce*I-induced indel mutations in mosquitoes scored as *kmo^R^*^G/Δ4^ in F_2_ (*B*), F_3_ (*C*), F_4_ (*D*) or F_5_ (*E*) generation. Delta (Δ) indicates nucleotide base deletion, and the plus mark (+) indicates the intact *DsRED* sequence. The indel-associated melting curve variants are shown in different colors.

Fig. S8. SSA-based transgene elimination interactions with homing and alternative applications. (*A*) SSA-based elimination of transgenes could result directly in a beneficial allele, such as recoding a mosquito protein to no longer serve as a host factor to pathogen transmission while maintaining its role in the vector. (*B*) Similarly, by positioning any cargo genes outside the direct repeats, SSA-based elimination would remove only the gene drive transgenes, leaving any anti-pathogen genes in place. (*C*) An individual heterozygous for the SSA/Gene drive (GD) transgene could undergo homing (down arrow) or SSA (up arrow) depending on where the DSB is induced. To prevent competition between the direct repeat during the homing step, an intervening sequence has been recoded to reduce the level of homology [green, recoded direct repeat (rDR)]. Otherwise, instead of homing-based gene drive, gene-drive resistant (GD-r) alleles would be produced directly, rather than through the SSA process, essentially short-circuiting the gene drive process.

Fig. S1.

Fig. S2.

Fig. S3.

Fig. S4.

Fig. S5.

Fig. S6.

Fig. S7.

Fig. S8.

| **Table S1. Generation of CRISPR/Cas9-driven transgenic lines.** | | | | | | | | | | | |
| --- | --- | --- | --- | --- | --- | --- | --- | --- | --- | --- | --- |
| Transgenic  mosquitoes | Donor DNAs | sgRNAs | Recipient strains | # Embryos injected | # G_0_ Larvae survived | # G_0_ outcrossed^a^ | | # G_1_ Larvae w/ phenotypes^b^ | | | |
|  |  |  |  |  |  |  |  | Blk | BlkG | WG | WGR |
| *kmo^EGFP^* | pBR-KmoEx4 | KmoEx4 | *Lvp* | ~2,000 | 125  (6.25%) | ♂51 | x *Lvp* | 12,045 | 78 (0.64%) |  |  |
|  |  |  |  |  |  | ♀60 |  |  |  |  |  |
| *kmo^RG^* | pSSA-KmoDR | HybRED | *kmo^EGFP^* | ~2,080 | 258  (12.4%) | ♂109 | x *kmo*^Δ4/Δ4^ |  |  | 14,949 | 32 (0.2%) |
|  |  |  |  |  |  | ♀126 |  |  |  |  |  |
| a, *kmo*^Δ4/Δ4^ is the TALEN-generated *kmo*-null mutant strain (40). b, Marker phenotypes: W, white eyes; Blk, black eyes; G, EGFP; R, DsRED. | | | | | | | | | | | |

| **Table S2. List of oligonucleotides for sgRNAs, PCR, and subcloning.** | |  |
| --- | --- | --- |
| Oligonucleotides | Sequences (5' to 3')^a^ |  |
| sgRNA-KmoEx4 | GAAATTAATACGACTCACTATAGG**ATGAATGTTCGGGTACTTCT**GTTTTAGAGCTAGAAA | |
| sgRNA-HybRED | GAAATTAATACGACTCACTATAGG**CGGTGCGGCCGCATAGGCGC**GTTTTAGAGCTAGAAA | |
| KmoEx4-F | TGTGAGTAGATTCCTTCGTCGTTGG |  |
| KmoEx4-R | ATTGCGTAGCAAGTTTACCTTGGGC |  |
| DmHsp70-F | AGCAAAGTGAACACGTCGCTAAGCG |  |
| DsRED-5Ra | TCACCTTCAGCTTCACGGTGTTGTGG |  |
| KMF2 | TTCTTCAAGACCAGGCCTCAATC |  |
| KMR1 | TCACTAAACTCAGCCAGTATCCTAT |  |
| Ex5-F1 | ACGACCGCATACAAAACGTACG |  |
| RED-3R | TCGTACTGCTCCACGATGG |  |
| SV40-F | AATCAGCCATACCACATTTGTAGAGG |  |
| In1-IR2 | AATCATGGGTAGGACGAATGTCTTACTCAGC |  |
| KmoHA1-F-Kpn | TTTTGGTACCGCCAGATCGCAGATAGAGTGTGC |  |
| KmoHA1-R-Age | TTTTACCGGTACCCGAACATTCATCTTTATTTC |  |
| KmoHA2-F-Av2 | TTTTCCTAGGCGGCCGCTAAAATAAACAACATTATCAG |  |
| KmoHA2-R-Av2 | TTTTCCTAGGGTTGGCTCTCTATTTGCACTCCACC |  |
| NosPro-F-M | GTCAACGCGTGGATCACTATCAAACCCCTAAGGAC |  |
| NosPro-R-B | GTCAGGATCCAGACATCCTCTAGATTTGTTCGTTGATC |  |
| SceI-F-B | GTCAGGATCCATGCCCAAGAAGAAGCGCAAGG |  |
| SceI-R-S | GTCAGTCGACTTATTTCAGGAAAGTTTCGGAGGAG |  |
| Nos3UTR-F-N | GTCAGCGGCCGCTCTAGACGTAATCGAAGTGTTGGAC |  |
| Nos3UTR-R-XR | GTCAGAATTCCTCGAGCGCCCTTTTCGTCATAAAATCGTAG |  |
| MRF1 | AAGACGATGAGTTCTACTGGCGTGGAATCC |  |
| MRR1 | CTTGCCGTATGTGATGGAGCGTTGTCATGG |  |
| MLF1 | TTGTTTACTCTCAGTGCAGTCAACATGTCG |  |
| MLR1 | TTCGACAGTCAAGGTTGACACTTCACAAGG |  |
| a, sgRNA target sequences are shown in bold letters. Restriction enzyme site sequences were underlined. | |  |

| **Table S3. Generation of *Mariner Mos1*-driven transgenic lines.** | | | | | | | |
| --- | --- | --- | --- | --- | --- | --- | --- |
| Transgenic mosquitoes | Donor Plasmids | Recipient strains^a^ | # Embryos injected | # Larvae survived | # G_0_ Adults  x *kmo*^Δ4/Δ4^ | # G_1_ Larvae w/ phenotypes^b^ | |
|  |  |  |  |  |  | W | WB |
| *Nos-I-SceI* | pMOS-3xP3-BFP-Nos-I-SceI | *kmo*^Δ4/Δ4^ | ~1,450 | 183  (12.6%) | ♂87 | 570 | 1  (0.18%) |
|  |  |  |  |  | ♀69 |  |  |
| *PUb-I-SceI* | pMOS-3xP3-BFP-PUb-I-SceI | *kmo*^Δ4/Δ4^ | ~1,150 | 263  (22.9%) | ♂107 | 1,704 | 96  (5.3%) |
|  |  |  |  |  | ♀116 |  |  |
| *β2T-I-SceI* | pMOS-3xP3-BFP-β2T-I-SceI | *kmo*^Δ4/Δ4^ | ~1,150 | 137  (11.9%) | ♂61 | 2,240 | 0 |
|  |  |  |  |  | ♀60 |  |  |
|  |  |  | ~1,600 | 240  (15%) | ♂128 | 19,253 | 0 |
|  |  |  |  |  | ♀98 |  |  |
|  |  |  | ~1,900 | 207  (10.9%) | ♂103 | 11,869 | 0 |
|  |  |  |  |  | ♀104 |  |  |
| *Hsp70A-I-SceI* | pMOS-3xP3-BFP-Hsp70A-I-SceI | *kmo*^Δ4/Δ4^ | ~1,600 | 111  (6.9%) | ♂58 | 10,357 | 0 |
|  |  |  |  |  | ♀41 |  |  |
|  |  |  | ~1,750 | 353  (20.2%) | ♂165 | 16,919 | 0 |
|  |  |  |  |  | ♀188 |  |  |
| a, *kmo*^Δ4/Δ4^ is the TALEN-generated *kmo*-null mutant strain (40). b, Marker phenotypes: W, white eyes; B, BFP. | | | | | | | |

| **Table S5. The single-generation test for SSA-based transgene elimination induced by the I-*Sce*I-expressing trigger strains (G_12_), *Nos-I-SceI* and *PUb-I-SceI*.** | | | | | | |
| --- | --- | --- | --- | --- | --- | --- |
| Parental cross (♂20 x ♀50) | Lineage of  SSA trigger (G_12_)^a^ | F_2_ Larval screening^b^ | | | | |
|  |  | # Total | # WGR No DSB (*kmo*^RG/Δ4^) | # WG  NHEJ (*kmo*^G/Δ4^) | # W *kmo*-null (*kmo*^Δ4/Δ4^) | # Blk  SSA (*kmo*^+/Δ4^) |
| *Nos-SceI* x  *kmo^RG^* | F_0_♂-F_1_♂ | 2000 | 990 | 3 | 1002 | 5 |
|  |  | 2080 | 1120 | 3 | 953 | 4 |
|  |  | 2240 | 1220 | 5 | 1004 | 11 |
|  | F_0_♂-F_1_♀ | 1380 | 700 | 1 | 675 | 4 |
|  |  | 1635 | 780 | 4 | 847 | 4 |
|  |  | 1350 | 661 | 4 | 677 | 8 |
|  | F_0_♀-F_1_♂ | 2340 | 1260 | 32 | 1035 | 13 |
|  |  | 980 | 490 | 10 | 473 | 7 |
|  |  | 2240 | 1140 | 25 | 1045 | 30 |
|  | F_0_♀-F_1_♀ | 1880 | 950 | 22 | 891 | 17 |
|  |  | 1240 | 673 | 13 | 546 | 8 |
|  |  | 2140 | 994 | 29 | 1103 | 14 |
| *PUb-SceI* x  *kmo^RG^* | F_0_♂-F_1_♂ | 6153 | 3075 | 2 | 3074 | 2 |
|  |  | 6630 | 3480 | 0 | 3150 | 0 |
|  |  | 9327 | 5170 | 0 | 4157 | 0 |
|  | F_0_♂-F_1_♀ | 1060 | 546 | 0 | 514 | 0 |
|  |  | 1570 | 954 | 0 | 616 | 0 |
|  |  | 1610 | 880 | 0 | 730 | 0 |
|  | F_0_♀-F_1_♂ | 269 | 139 | 0 | 130 | 0 |
|  |  | 1416 | 678 | 0 | 738 | 0 |
|  |  | 2280 | 1310 | 0 | 970 | 0 |
|  | F_0_♀-F_1_♀ | 2398 | 1300 | 0 | 1098 | 0 |
|  |  | 2080 | 1100 | 0 | 980 | 0 |
|  |  | 1783 | 927 | 0 | 856 | 0 |
| a: *nos*-driven germline cell-specific expression of the homing endonuclease, I-*Sce*I. b: W, white eye; Blk, black eye; G, EGFP; R, DsRED; B, BFP. | | | | | | |
